# A key role of the basal ganglia in pain and analgesia - insights gained through human functional imaging

**DOI:** 10.1186/1744-8069-6-27

**Published:** 2010-05-13

**Authors:** David Borsook, Jaymin Upadhyay, Eric H Chudler, Lino Becerra

**Affiliations:** 1 P.A.I.N. Group, Harvard Medical School, 115 Mill Street, Belmont, MA 02478, USA; 2 Department of Anesthesiology, University of Washington, Box 356540, Seattle, WA 98195, USA

## Abstract

The basal ganglia (BG) are composed of several nuclei involved in neural processing related to the execution of motor, cognitive and emotional activities. Preclinical and clinical data have implicated a role for these structures in pain processing. Recently neuroimaging has added important information on BG activation in conditions of acute pain, chronic pain and as a result of drug effects. Our current understanding of alterations in cortical and sub-cortical regions in pain suggests that the BG are uniquely involved in thalamo-cortico-BG loops to integrate many aspects of pain. These include the integration of motor, emotional, autonomic and cognitive responses to pain.

## Introduction

**Figure 1:**
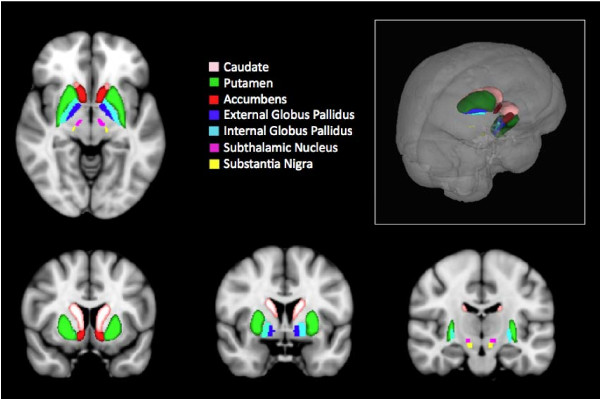
**Basal Ganglia**. Horizontal (A), Coronal (B-D) and (E) 3-D representation of the Basal Ganglia in the Human Brain. Sub-regions are noted in the color-coded key. Brain sections from FMRIB Software Library http://www.fmrib.ox.ac.uk/fsl/.

The basal ganglia (BG) consist of the striatum (caudate (C), putamen (Pu) and the core of the nucleus accumbens), the external segment of the globus pallidus (GP), the internal segment of the globus pallidus (GPi), the subthalamic nucleus (STh), and the substantia nigra (SN) [1-3] (Figure 1). Although best known for their role in motor systems, these regions are a major site for adaptive plasticity in the brain, affecting a broad range of normal behaviors [4] and neurological and psychiatric conditions [5].

The first section of this review, *Basal Ganglia: Pain Processing*, summarizes the role of the BG in pain processing as has been reported in specific research and review papers. In the section titled *Basal Ganglia: Functional Imaging Studies of Pain in Humans*, we describe functional imaging studies in humans reporting activation of the BG in acute pain, chronic pain, and in response to analgesics. The third section titled *Basal Ganglia: Lessons Learned from Functional Imaging *attempts to integrate the information about lessons learned from functional imaging. The final section, *Basal Ganglia: Imaging and Improved understanding of Clinical Applications*, describes how imaging may provide insight to how and why certain therapies may be useful. Future measures of BG function may contribute significantly to our understanding about the brain changes associated with chronic pain and specific therapies that may change the brain in a manner that corresponds with therapeutic effects.

### Basal Ganglia: Pain Processing

Both preclinical and clinical data support a role for the BG in pain processing [6,7].

Data supporting a role for the BG in pain and analgesia processing have been derived from numerous preclinical studies. These studies include electrophysiology [8,9], analgesic effects of microinjections into these regions [10], electrolytic lesion studies [11], chemical lesions of dopaminergic terminals [12,13], activation of striatal dopamine systems producing analgesia in rats [14] and imaging drug effects in neuropathic rat models [15]. In addition, novel pain pathways, for example those projecting from the spinal cord to the globus pallidus [16], have been discovered. Since the major review on the involvement of the basal ganglia in pain [6,7], the examples of preclinical studies noted above are not exhaustive and more recent studies have included numerous other contributions (e.g., [9,10,17-19]).

In the clinical domain, two disease patterns epitomize the role of the BG in pain - Parkinson's disease (PD) and Complex Regional Pain Syndrome (CRPS). In PD, the initial pathology involves the BG (i.e., loss of dopaminergic neurons in the substantia nigra [20]) that results in movement disorders, and affected subjects frequently have chronic pain [21-24]. In CRPS the initial event is most often relatively minor damage to a peripheral nerve [25,26], but with time expression of frequently associated movement disorders may become evident, thereby implicating BG involvement [27-30]. Both conditions are associated with movement disorders and both are associated with pain. In chronic pain, alterations of multiple sub-cortical and cortical processing, including sensory, emotional/affective, cognitive and modulatory systems, are present. Independent of specific somatosensory regions and pain modulation (e.g., SI and pain intensity, PAG and pain facilitation/inhibition), recent functional neuroimaging data suggest that the BG appear to be intimately involved in these processes.

Lesions of the BG in patients have offered further insights into the potential role of BG in pain and analgesia. Infarction of the lenticular nucleus (composed of the putamen and globus pallidus) may result in sensory deficits including pain in some patients [31]. Both unilateral and bilateral deep brain stimulation of the globus pallidus have been reported to improve pain by approximately 70%, and this improvement may persist for a significant period of time [32]. In Parkinson's disease, bilateral pallidotomy produces pain relief [33]. Taken together, these data provide insights into the potential contributions of the BG in chronic pain in humans.

**Figure 2:**
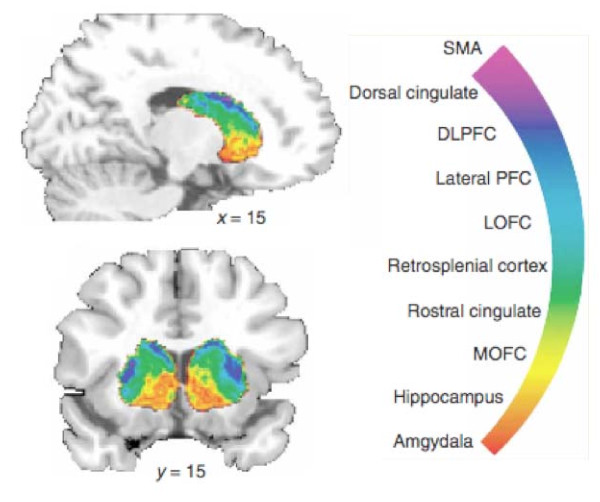
**Cortical Connectivity and Basal Ganglia**. The BG receive multiple inputs from cortical and subcortical regions as noted in the figure. Many of these regions are involved in pain processing (see text). (From [175], Nature Neuroscience, Nature Publishing Group, with permission).

Multisensory integration takes place in the BG [34,35] that serves an important role in behavioral actions including motor responses [36]. The BG receives inputs from all cortical areas (including medial and orbital, prefrontal, dorsolateral, premotor and motor cortex, sensorimotor and parietal cortex) and the thalamus. Multiple cortical areas receive afferents from a single thalamic nucleus and send back information to different thalamic nuclei [37]. Efferent pathways from the BG project to frontal lobe areas including prefrontal, premotor and supplementary motor areas (used in motor planning). Thus the BG are well positioned to have influences on cortical regions involved in motor responses, in behavior relating to predicting events, and involved in attention and learning [38]. Using probabilistic tractography, a recent diffusion tensor imaging (DTI) study on connectivity patterns of the BG in humans [39] showed segregated and overlapping loops that include prefrontal, premotor, and motor networks to BG sub-regions (see Figure 2). In the cat for example, electrophysiological recordings have shown that neurons in the BG respond to visual, auditory and somatosensory information [35]. How this integration takes place is not well understood, but BG circuits are in a unique position to integrate cortical information to increase the speed of data processing tasks [40-42]. Similar processing may take place with emotional and cognitive information. Such processing is relevant to pain because the response to acute or chronic pain involves sensory, motor, autonomic, cognitive and emotional integration.

**Figure 3:**
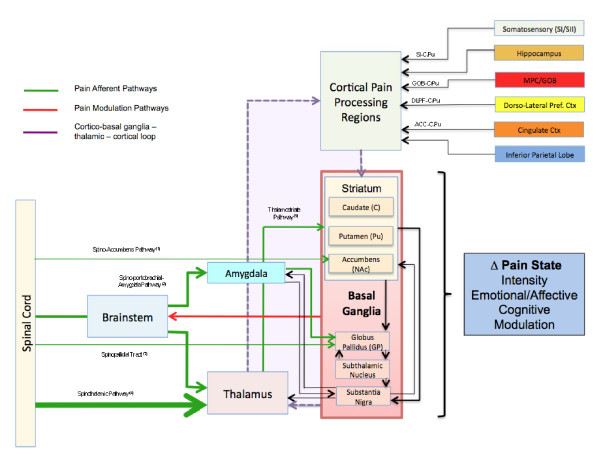
**Basal Ganglia and Pain Systems**. Afferent inputs from spinal cord and brainstem have direct and indirect inputs into the BG, most are into the striatum but some input into the globus pallidus and substantia nigra. Thalamic inputs into various cortical regions are then processed and complete the cortico-BG-thalamic loop. Cortical inputs include those from a number of regions known to be involved in pain processing. **Key**: (1) Spino-accumbens pathway [57]; (2) Spino-parabrachial-amygdala pathways [176]; (3) Spino-thalamic pathway [177]; (4) Thalamo-Striatal pathway [38].

Figure 3 shows the connectivity patterns of the BG with respect to possible pain processing. Pain inputs into the BG may be considered to be from two major sources: (1) Afferent inputs from pain sensing systems via direct (e.g., spino-BG) and indirect (e.g., spino-thalamic-BG) pathways; and (2) from cortical and sub-cortical brain regions that contribute to the BG-Thalamic-Cortical loops. Cortical regions involved in these feedback loops are also known to have important roles in pain processing. These areas include the ACC, regions of the frontal lobe (e.g., dorsolateral prefrontal cortex (DLPF), orbitofrontal cortex (Gob)), parietal, insular, and hippocampal regions. Chudler and Dong have also reported putative nociceptive pathways into and out of the basal ganglia [6,7].

### Basal Ganglia: Functional Imaging Studies of Pain in Humans

Functional imaging studies in healthy volunteers and patients with chronic pain have supported a growing role of the BG in pain processing. Previous work has suggested that the BG may be involved in most aspects of pain processing including sensory-discriminative, emotional/affective, cognitive dimension of pain and pain modulation [7]. Given our current understanding of these brain regions in the pain brain phenotype (i.e., functional brain changes in healthy and disease (see [43]) and our understanding of the basic integrative role the BG may play neural processing, it is suggested that the BG play a pivotal role in the behavioral manifestations of chronic pain. Although a large literature exists on the role of the BG in non-motor activities [44], brain imaging studies of acute and chronic pain have contributed to supporting preclinical and clinical work as playing an increasingly important role in acute and chronic pain processing and in the effects of some analgesics on brain function (Tables 1, 2 and 3).

**Table 1:** fMRI Studies of Pain and Basal Ganglia Activation.

Study Type	Condition	Stimulus	C	Pu	NAc	GP	STN	SN	Reference
**Acute Pain**									

	Thermal Pain	Contact Heat	+	**+**	-	**+**		**+**	[45]

		Contact Heat			-				[142]

		Contact Heat			-				[58]

		Contact Heat		**+**					[143]

		Contact Heat	+						[144]

		Contact Heat	+						[145]

		Contact Heat	+	**+**					[146]

		Contact Heat		**+**					[47]

		Contact Heat						**+**	[147]

		Laser		**+**					[148]

		Cold	+						[49]

		Cold (Prickle)	+						[48]

	Electrical Pain	Current	+						[51]

		Current		**+**					[95]

		Electroacupuncture			**+**				[149]

	Pressure Pain		+	**+**		**+**			[150]

	Capsaicin Sensitization	Punctate Mechanical		**+**					[91]

		Punctate Mechanical		**+**	**(+)**	**+**		**+**	[151]

	Visceral Pain	Fundus Distention	+	**+**					[152]

		Esophageal Distention	+	**+**					[143]

**Chronic Pain**									

	Neuropathic	Trigeminal (cold allodynia)	-			**+**			[56]

		Trigeminal (mechanical allodynia)	-	**+**	**+**	**+**			[56]

		Post Herpetic (spontaneous pain)		**+**	**(+)**				[153]

	Complex Regional Pain Syndrome	Pediatric	-						[70]

	Fibromyalgia								[154]

		Pressure		**+**					[150]

		Catastrophising		**+**		**+**			[155]

	Back Pain				**+**				[156]

	Osteoarthritis			**+**					[156]

	Visceral	Irritable Bowel Syndrome	-	**+**					[157]

**Empathy**									

	Visual								

		Pictures	+	**+**					[158]

		Pictures	+						[159]

		Virtual Pain (needles)		**+**					[160]

**Analgesics (phMRI)**									

	Opioid Agonists	Morphine		**+**	**+**			**+**	[58]

		Remifentanil	**+**	**+**		**+**		**+**	[83]

	Opioid Antagonists	Naloxone	**+**		**+**		**+**	**+**	[82]

**Key**: E = evoked stimuli; + = increased BOLD signal; - = decreased BOLD signal

**Table 2:** fMRI Measures of the Effects of Analgesics on Pain.

Study Type	Condition	Stimulus	C	Pu	NAc	GP	STN	SN	Reference
**Analgesic Effects on Pain**									

Acute Pain									

	Capsaicin	Gabapentin	**(-)**						[91]

	Heat Pain	Naloxone	**+**	**+**					[82]

**Table 3:** Positron Emission Tomography (PET) studies of Pain.

Study Type	Condition	Receptor Binding	C	Pu	NAc	GP	STN	SN	Reference
**Acute Pain**									

	Thermal Pain	Contact Heat (15)O-water		**+**					[161]

		Contact Heat (15)O-water		**+**					[162]

		Contact Heat (15)O-water		**+**					[163]

	Contact Heat (Pain Threshold)	Dopamine receptor binding (11C) Raclopride		Binding - inverse correlation to Heat thershold					[133]

	Muscle Pain Hypertonic Saline	(15)O-water		**+**					[164]

		Dopamine Receptor Binding (11C) Raclopride	+	+	+				[135]

	Visceral Pain	Gastric Distenton (15)O-water	+						[165]

Placebo									

	Muscle Pain Hypertonic Saline	Opioid and Dopamine receptor binding Placebo (11C) Raclopride (11C) Carfentanil			Increased opioid and dopamine release				[65]

		Opioid and Dopamine receptor binding Placebo (11C) Raclopride (11C) Carfentanil			Decrease opioid and dopamine release				[65]

**Chronic Pain**									

	Neuropathic Pain (Burning Mouth)	6-[(18)F]fluorodopa		Decreased presynaptic dopamine function					[72]

	Neuropathic Pain	Motor Cortex Stimulation (15)O-water		**+**					[141]

	Atypical Facial Pain	Dopamine receptor binding [11C]raclopride		Increased D2 binding					[73]

	Fibromyalgia	μ receptor (11C) Carfentanil			Decreased Opioid Binding				[166]

		Dopamine receptor (11C) Raclopride			Decrease dopamine release				[74]

**Analgesics**									

	Fentanyl	Opioid Effects (15)O-water	+						[167]

	**Ketamine**	NMDA Receptor Binding (n-methyl11C)-ketamine	+	**+**					[168]

Since there are multiple sub-regions within the BG, preclinical studies using specific methods cannot easily define how these interact at the same time to integrate such information, including pain. Understanding the functional processing of pain and analgesia in these regions might afford insights into complex behaviors of brain systems in acute and chronic pain and help us understand how analgesics might affect pain processing. Brain imaging in humans includes a number of methods such as functional MRI (fMRI), pharmacological MRI (phMRI), morphometric/anatomical measures (diffusion tensor imaging and gray matter volumetric analysis), and positron emission tomography (functional PET (fPET) and ligand binding/displacement (phPET)) that allow whole brain evaluation of specific circuits.

### Basal Ganglia Activation in Acute Pain (Figure 4, Tables 1, 2 and 3)

**Figure 4:**
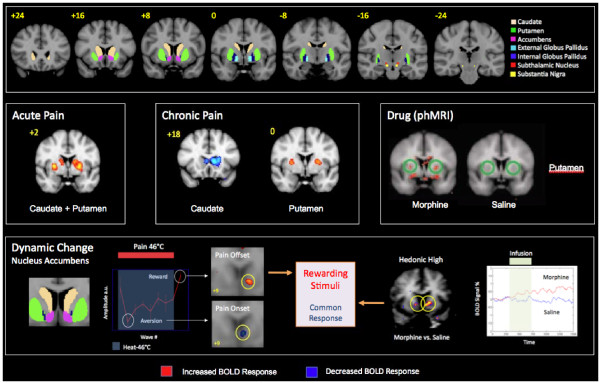
**Examples of Basal Ganglia Activation in Pain and Analgesia**. fMRI BOLD Activation in the nucleus accumbens following acute pain, allodynia neuropathic pain to cold and brush, and to morphine.

#### Noxious Stimuli

Early studies of pain using fMRI indicated activation patterns in the BG including the putamen and globus pallidus. These early observations have now been replicated in a number of functional imaging studies of experimental pain in humans, examples of which are shown in Tables 1 and 2. As noted in the tables, a considerable similarity exists in the activation patterns from multiple stimuli, including thermal (heat and cold), mechanical (in a hyperalgesic capsaicin model), painful electrical and visceral pain. Subsequent studies showed what seemed to be specific regional activation in other BG structures including the nucleus accumbens [45] and putamen [46,47]. However, most studies have evaluated or reported activation patterns and observed striatal involvement in a general context.

Tables 1 and 2 show positive activation is present in the caudate nucleus across multiple studies and across multiple modalities. In addition to those mentioned in the tables, activation in the caudate is present in "prickle" sensation evoked by cold stimuli [48]. In our own studies, cold has previously been shown to activate basal ganglia including the caudate [49]. Activation in the caudate nucleus to noxious stimulation has been suggested to be part of a pain modulatory system [50,51]. In support of this, electrical stimulation of the caudate in non-human primates diminished pain reactivity [52]. The authors suggested that the results indicate that the effect of caudate stimulation is to reduce the affective components of pain elicited by noxious electrocutaneous stimuli.

A few studies have evaluated specific activation in the putamen and nucleus accumbens. As noted in Tables 1 and 2, the putamen is commonly activated across most acute pain imaging studies. Pain activated the putamen bilaterally and a somatotopic organization for hand- and foot-related responses was only present in the contralateral putamen [53]. In healthy women, pain-evoked putaminal activation occurred during their follicular phase [54].

Activation of globus pallidus following painful stimulation has been shown in healthy subjects (Tables 1 and 2). Unlike the caudate and putamen, fewer studies have specifically reported activation in this BG structure. However, like the caudate and putamen, the globus pallidus has neurons that respond to noxious stimulation [9].

The nucleus accumbens (NAc), a brain substrate known to be involved in reward-aversion processing, has been shown to respond with opposite BOLD signal valence to rewarding or aversive stimuli [45,55]. Using a pain onset (aversive) and pain offset (rewarding) prolonged stimulus, a negative signal change with pain onset and a positive signal change with pain offset was observed in the NAc contralateral to the stimulus. The study supports the idea that the NAc fMRI signal may provide a useful marker for the effects of pain and analgesia in healthy volunteers. A parallel study on NAc activation in response to the direct effects of the analgesic (pain offset) morphine, showed the same increased (rewarding) BOLD signal in the structure [56]. Direct afferents from the trigeminal nucleus to the nucleus accumbens have been demonstrated in the rat; these have contralateral projections from lamina I but bilateral with contralateral predominance from lamina V [57]. These anatomical studies seem to agree with other studies showing contralateral activation in the NAc [45,55]. Subsequent studies indicated two components of activation within the NAc - an anterior, superior, and lateral component and another component that was posterior, inferior, and medial within the structure. The anatomical segregation may correlate with the functional components of the NAc (i.e., shell and core) that have been defined in other species (see [58]). The results support heterogeneity of function within the NAc and have implications for understanding the contribution of NAc function in processing of pain and analgesia [58]. The NAc has a pivotal role in aversive and rewarding (hedonic) processing [59].

Given that the reward system is part of the pain network [45], and that the placebo response is clearly involved in analgesia [60,61], it is not surprising that functional imaging has been the ideal technology to better understand neural networks involved in placebo in humans [62,63]. Recent reviews suggest that multiple brain regions (e.g., anterior cingulate cortex, anterior insula, prefrontal cortex and periaqueductal grey) are important in the placebo response [64]. Other studies strongly implicate the NAc and ventral BG in the placebo response [65].

### Basal Ganglia Activation in Chronic Pain (Figure 4, Tables 1, 2 and 3)

An increase in gray matter in the BG has been reported in chronic back pain patients [66], in fibromyalgia [67], and in chronic vulval pain [68], suggesting some underlying functional alterations that drive these changes. In other chronic pain conditions, alterations in activation in BG regions have been demonstrated [55,69] where stimulation to the affected neuropathic regions produces significant activation in the BG. Specifically, contrast analysis between the affected and unaffected regions resulted in increased activation in the globus pallidus and putamen to mechanical and cold allodynia, as well as decreased activation in the caudate nucleus to mechanical, cold and heat allodynia [55]. Taken together these data appear to show a consistent decrease in the caudate nucleus to pain produced across multiple painful stimuli. Similarly, functional imaging of pain in a group of pediatric patients with CRPS has shown significant activation in the BG with cold and brush stimuli, and also displays decreased activation in the caudate nucleus [70].

Chronic pain has also been shown to alter BG structure with measures of gray matter volume and white matter tract integrity. In a more recent report, measures of gray matter morphometry and white matter anisotropy were measured in patients with complex regional pain syndrome (CRPS) [71]. Alterations in morphometry indicated atrophy in a single cluster encompassing the right insula, right ventromedial prefrontal cortex (VMPFC), and right NAc. Additionally, decreased connectivity was reported between the ventromedial prefrontal cortices to the BG [71].

Alterations in dopaminergic and opioidergic function in the BG have been reported in clinical pain conditions. In patients with burning mouth syndrome, decreases in dopamine in the putamen suggest reduced dopaminergic inhibition may contribute to the chronic pain condition [72]. In addition, both atypical facial pain [73] and fibromyalgia patients [74] show an abnormal dopamine response. Such data are consistent with other conditions associated with decreased dopamine, including Parkinson's disease [75,76]. In addition to the alterations in dopamine, decreased μ binding in chronic pain conditions such as fibromyalgia may contribute to altered pain processing [77]. In psychiatric disorders, pain produces increased activation in putaminal regions in post traumatic stress disorder, or PTSD, [78] that when considered with the diminished pain sensitivity reported in this patient group may also be caused in part to abnormalities within the BG.

### Basal Ganglia Activation and Analgesics (Figure 4, Tables 1, 2 and 3)

Functional imaging of drug effects (also known as pharmacological MRI or phMRI) has been done in the context of BG function in preclinical [79] and clinical [80,81] conditions. The evaluation of direct brain changes in functional activity in response to known analgesic drugs has been limited to a small group that include morphine [55], naloxone [82], remifentanil [83] and buprenorphine [84]. Direct effects of μ opioid agonist or antagonist infusions both show activation in the BG, including the NAc. In these experiments, the direction of BOLD signal is opposite with increased activation following morphine infusion [55] and a decreased activation following infusion of naloxone [82] in healthy volunteers. The opposite direct pharmacological effects indicate some specificity of response but may also underlie NAc drive with respect to pain and analgesia. An increased activation may be part of an endogenous analgesic drive or emotional drive since increased activation patterns within the NAc are also seen in other fMRI experiments on reward [85-87]. The increase (hyperalgesic) pain response (inhibition of tonic opioid drive) to pain was observed in brain regions following naloxone that included concurrent activation in the globus pallidus, caudate and putamen. Opioids may produce changes in the BG including increases in D2 receptor binding [88]. However not all imaging studies report BG activation in response to opioids, either because they were not specifically focused on these regions or they used less sensitive methods (e.g., alfentanil [89]; remifentanil [90]).

Few authors have evaluated the effects of non-opioid analgesics on BG responsiveness. In one example, although not specifically referred to in their paper, gabapentin reduced activation in the BG [91]; specifically, for gabapentin vs. placebo comparison in a hyperalgesic paradigm, deactivation in the caudate was present in the single dose study in healthy volunteers.

Other drugs, considered to be analgesics, have been evaluated using imaging in a similar manner to that noted above, and these studies report alterations in the human striatum. For example tetrahydrocannabinol (known to be an analgesic [92]) produces dopamine release in the striatum [93]. Similar results have been shown for the NMDA antagonist ketamine (reviewed by [94]).

### Lessons Learned from Imaging

Based on imaging data of pain as well as a growing literature on pain in conditions with abnormalities of BG function, it is clear that a constellation of brain regions plays an important role in acute and chronic pain (see Tables 1, 2 and 3). With the exception of studies on the NAc and the pallidum (see above), few functional imaging studies in humans have attempted to evaluate other regions of the BG function in pain. Nevertheless there is increasing evidence for the important roles of the region in neural systems' adaptation to pain.

#### Putamen (P) shows consistent increase in activation across multiple pain imaging studies

The putamen, a structure that shows somatotopic activation to pain [53], is consistently activated during acute and chronic pain conditions and is affected by analgesic administration. Chronic pain has also been shown to increase putamen volume (see [67]) suggesting, perhaps, that a continuous drive producing increased activation may result in processes that enhance gray matter volume in the structure. This region may receive direct inputs from sensory systems, but also from cortical inputs. Some studies (e.g., [83]) have suggested delayed functional activation in BG circuits (e.g., caudate) following remifentanil, implicating that activation in these regions occurs as a consequence or subsequent to initial activation of thalamo-cortical circuits. Activation seems to be present with painful stimuli but missing with non-painful stimuli [95].

#### Opposite Signal Changes in Caudate and NAc in Acute vs. Chronic Pain

In contrast to increases in putaminal activation for acute pain, BOLD signals are opposite in chronic pain across multiple pain studies. The reason for this observation is not known although the nature of reward processing in acute vs. chronic pain is clearly different [45,59,71].

#### Smaller Nuclei (ST, SN) are not clearly defined in many fMRI studies

Only a few brain imaging studies have reported activation in the smaller nuclei of the BG even though they may be present. Although there are studies in animals investigating SN mechanisms of pain [96], future brain imaging studies in humans will contribute to our understanding of the role of these nuclei in pain processing. Developments such as accurate automated identification of sub-nuclei within the basal ganglia should be helpful in measures of functional brain changes [97].

#### Feed-forward and Feedback Loops - Integrating, Modifying, and Modulating the Pain Experience

The BG are involved in the integration of information between cortical and thalamic regions and in particular the three domains of pain processing - sensory, emotional/cognitive and endogenous/modulatory. Some investigators have suggested that these regions have evolved as a "centralized selection device to resolve conflicts over access to limited motor and cognitive resources" [98]. Phylogenetically older, sub-cortical connections exist between the BG and brainstem regions involved in sensorimotor processing [99], and more recent evidence points to BG involvement through direct connections from sensory inputs not involving cortical loops [100]. Dysfunctional cortico-BG-thalamic loops may contribute to the maintenance of chronic pain and the evolution of altered neural processing that may be a basis for co-morbid behaviors. Whether brain processing of pain is dependent on external neural drive (i.e., peripheral/feed-forward inputs) or internal neural drive (i.e., subcortical or cortical drive/feedback), the BG are central processors that may play a role in integrating these divergent inputs that may modify pain over time.

### Basal Ganglia: Imaging and Improved Understanding of Clinical Applications

#### Cortical Stimulation and Chronic Pain - Acting via Cortico-Striatal-Thalamic Loops

Cortical stimulation has been used either directly with electrodes placed on the motor cortex [101] or through the use of repetitive transcranial stimulation (rTMS) [102]. Some investigators have postulated that motor cortex neurostimulation produces an analgesic effect by modulation of the affective components of pain and not of the sensory components [103]. In support of this hypothesis, stimulation of the secondary somatosensory cortex (SII), but not motor cortex, results in increased pain thresholds and altered discriminative capacity to pain [104]. A combination of motor cortex stimulation (MCS) and postoperative fMRI showed an inhibiting effect on the primary sensorimotor cortex as well as on the contralateral primary motor and sensitive cortices [105] without changes in BG. Others have argued that MCS may act through activation of perigenual cingulate and orbitofrontal areas to alter the emotional appraisal of pain or cortical-brainstem (e.g., PAG) activation that enhances inhibition of pain [106] or alterations in endogenous opioid systems [107]. Given the cortico-striatal loops, stimulation of a number of cortical regions may thus be involved in pain reduction. Stimulation of dorsolateral prefrontal cortex, motor cortex or sensory cortices may have different effects based on the specificity of cortico-striatal loops. In addition, connectivity between the BG and modulatory regions including the PAG/brainstem or through the NAc [108-110] may also contribute to the potential mechanism of cortical activation resulting in analgesia.

#### Anterior Cingulate Lesions and Pain Control

Both clinical [111] and preclinical [112] studies have suggested that the ACC results in alterations of pain processing. The interesting issue seems to be the dissociation of pain intensity from pain affect (caring about the pain) [113,114]. The ACC in humans may be involved with linking reward-related information [115] and with alternative actions, since destruction of the ACC results in errors related to planning [116]. The ACC has projections to the caudate nucleus and the NAc [117], and ACC lesions may produce an attenuation of a negative pain affect [118] through a combination of neural networks that include these circuits.

#### BG and Learned Behaviors

Pain is clearly a complex process that affects multiple brain systems. It debatable if pain is a learned behavior, but the BG may have a role in learning due to its involvement in habit and stimulus-response learning [119]. Such learning may be derived from pain related regions involved in sensory (e.g., pain intensity coding regions such as SI), affective (e.g., cingulate or anterior insula) or cognitive functioning (medial and lateral prefrontal cortices). Similar to the notion of "chunking action repertoires" for motor action, it may be that pain related repertoires include motor related changes.

#### BG and Emergence of Central Pain

Aside from CRPS, other conditions including depression and Parkinson's disease have BG pathology that may be important in their clinical presentation of centralized pain [120]. In Parkinson's disease with central pain, electrophysiological measures of pain pathways are normal, but hyperalgesia to repetitive stimuli is present; this is attenuated by L-dopa [121], and altered central processing in these conditions results in generalized pain symptoms. Although the basis for these changes is unknown, it may be the result of altered chemicals in the BG, such as central dopamine, or related to aberrant networks that induce a kindling-like pain [122]. Changes in neural connectivity that produce changes in increased synaptic strength [123] and could include pain facilitatory circuits [124] may also underlie these changes. Although the basis for these changes is unknown, alterations may then lead to other changes such as abnormal gating of sensory-motor function involving parallel changes thalamic regions [125]. Imaging patients with depression in whom non-dermatomal sensory deficits were present shows that hypometabolic patterns (FDG-PET) of activation in the putamen was observed [126].

Regions of the thalamus including the paramedian and anterolateral are known to be involved with central pain (e.g., post stroke pain) [38,127]. The striatum receives excitatory input from the thalamus. The centromedian (CM) and parafascicular (Pf) thalamic nuclei are important sources of thalamostriatal projections [128] and send connections to the putamen and caudate [128,129]. Moreover, the topography of these connections corresponds with cortical sensorimotor territory observed following cortical injections [130].

#### Basal Ganglia and Opioid Systems

The BG has high levels of endogenous opioids, and high binding of opioid receptors are present within the BG [131]. In a number of chronic pain conditions, receptor-binding studies indicate a decreased opioidergic tone. Many analgesics act at the level of the basal ganglia and may contribute to both analgesic and addictive processes. An understanding of how these functional processes are differentiated by different endogenous chemicals (e.g., opioidergic vs. dopaminergic (see [65])) may contribute to better analgesics since opioidergic tone might be abnormal [77].

#### Dopaminergic Drugs and Pain

Given that pain now has a functional basis in terms of regions activated including those that are classically involved in reward [45], and that chronic pain may be a reward deficit syndrome [132], modulating dopamine may have important possibilities for pain treatment. Parkinson's disease patients have improved pain control when treated with L-dopa [21,121]. Changes in dopamine are a critical element in Parkinson's disease where abnormal pain processing is present in both conditions. Recent imaging data have reported a direct correlation between striatal dopamine D2/D3 receptors and sensory thresholds as being selective for the modality of pain but not for non-painful stimuli [133]. Striatal dopamine D2/D3 receptors may control a modulatory pathway producing a parallel shift in the stimulus-response function for sensory signals [134]. Others have suggested differential processing of pain within the BG - a more dorsal DA D2 receptor-mediated neurotransmission in the caudate and putamen that correlates with subjective ratings of sensory and affective qualities of the pain, along with a more ventral system involving the NAc, is associated with emotional processing [135]. Such differences may be important in drug effects on pain. The use of antipsychotic medications for pain is not new [136], and the number needed to treat (NNT; this is the number of patients to be treated for the first subject to show a 50% analgesic effect) of 2.6 is very competitive with the best drugs available for chronic pain [137]. However, these drugs have extrapyramidal and sedative effects, and atypical antipsychotics that have fewer side effects may have analgesic properties as assessed in a limited number of studies [138]. Newer drugs that target specific dopamine receptors may prove to be more useful.

#### BG Deep Brain Stimulation and Strategies for Pain Relief

Brain imaging affords the possibility to measure changes in brain circuits that may be altered as a result of deep brain stimulation. Some of these have shown specific changes in BG circuitry [139]. In the latter, stimulation of the ventroposterior medial thalamus (VPM) resulted in decreases in activation of the substantia nigra when activation prior to stimulation vs. post stimulation that provided pain relief was measured (pain, no stimulation vs. no pain, no stimulation). Thalamic stimulation may activate thalamo-cortical-BG loops that contribute to the analgesic response [140].

## Conclusions

**Figure 5:**
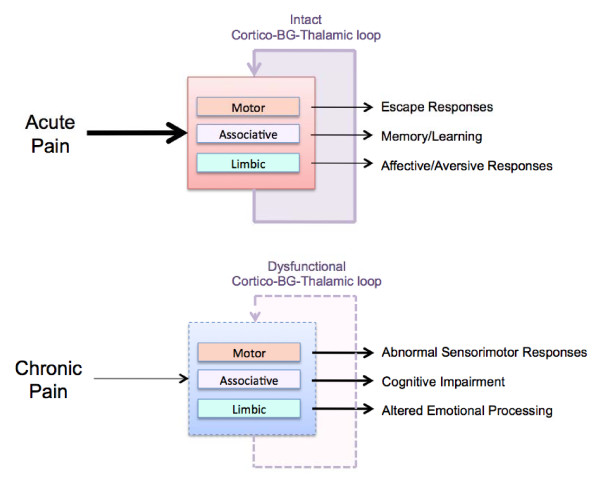
**Basal Ganglia Function in Acute and Chronic Pain**. **Top**: Acute Pain Processing is a normal response where pain produces responses in brain circuits that usually revert to normal. Some of these processes are integrated in the BG and result in escape responses: components of memory and learning of pain and affective responses to pain. **Bottom**: In chronic pain both inputs from peripheral systems and cortical and subcortical regions are abnormal. The result is that BG functions as well as cortico-BG-thalamic loop functions are altered. The result may be altered integration of sensori-motor responses, cognitive impairment and emotional processing.

Functional imaging of pain has shown clear and consistent changes in the BG in pain conditions. Table 4 summarizes the salient features of each sub-region of the BG as it pertains to overall putative function and specific functions in pain, and Figure 5 summarizes potential alterations in BG outputs affecting behaviors in acute and chronic pain. Future work should help contribute to further understanding functional and anatomical connectivity of inputs and circuits that show how the BG may be involved in acute and chronic pain. Such findings may present an increasing and important role of these brain regions in the centralization of chronic pain and the contribution to the altered brain in chronic pain conditions. Future studies, using a combination of imaging approaches, will help define the specificity of BG in pain processing. For example, functional connectivity analysis can demonstrate probable correlations between BG subdivisions and other brain regions [141].

**Table 4:** Components of the Basal Ganglia - Putative Function in Pain Processing.

Region	Putative Role in Pain	Reference
Caudate	Involved in avoidance behavior to pain	[169]
	Decrease pain sensitivity following apomorphine injections	[14]
	Encode noxious stimuli intensity to minimize bodily harm	[9]
	Behavioral Reinforcement (? Including Pain)	[3]

Putamen	Somatotopic modulation of pain	[148]
	Variations in subjective ratings of pain	[135]

Nucleus Accumbens	Affective Valence for Reward and Aversive Stimuli	[45,170]
	Processing of emotional salience of pain	[135]

Globus Pallidus	Encoding of behavioral repertoires (? Including pain)	[3]
	Deep brain stimulation inhibits pain	[89,171]
	Morphine analgesia	[172]

Subthalamic Nucleus	Functional suppression of neural messages	[173]
	Behavioral Inhibition	[174]
	Regulates level of execution of cortical commands; processing of emotional, cognitive and motor behavior	[3]

Substantia Nigra	Heterogeneous response to aversive stimuli	[96]

## Competing interests

The authors declare that they have no competing interests.

## Authors' contributions

DB and JU conceptualized the paper. All authors contributed to the drafting of the paper. All authors have read and approved the final manuscript.
